# Rapid Methods of Early Diagnosis of Renal Tuberculosis*Read before Georgetown University Clinical Society. January 27, 1917—Reprinted from The Virginia Medical Semi-Monthly.

**Published:** 1917-08

**Authors:** J. C. Blakistone

**Affiliations:** Washington, D. C.; Genito-Urinary Surgeon Casualty Hospital & Washington Asylum Hospital


### RAPID METHODS OF EARLY DIAGNOSIS OF RENAL TUBERCULOSIS.*

**By J. C. Blakistone, M. D., Washington, D. C.**
Genito-Urinary Surgeon Casualty Hospital & Washington Asylum Hospital.

In the last three years I have had referred to me fourteen cases of renal tuberculosis; of these, I have removed the kidney in four cases. All of these cases have been studied at some length, all have had careful cystoscopic examination; microscopic examination of the urine has been made, and rather lengthy histories taken. Besides these I have examined almost fifty specimens of urine from either real or suspected cases of renal tuberculosis; of these, I have removed the kideral specimens from the same case. In looking over the histories of these cases I have been much impressed by the fact that there are no symptoms of the disease which could be considered characteristic; that in so far as my cases have shown from a detailed study of the symptoms, I was not enabled to even infer the cause of the trouble before more exact methods of diagnosis were undertaken except in the very late cases.

By this is meant that, though practically all my cases showed such symptoms as bladder irritability, painful and frequent urination, a tendency to polyuria, an acid urine, pus and at times blood in the urine, yet these symptoms and signs in themselves were not such as to distinguish the infection from other infections of the genito-urinary tract. In none of my comparatively early cases were there symptoms of pain in the kidney region, and even in the late cases pain in this region was an infrequent symptom. Though loss of weight and failure of general health were present to some extent in all my cases, yet it is remarkable how little the general health may be impaired in the early stage of the disease.

---

*Read before Georgetown University Clinical Society, January 27, 1917—Reprinted from The Virginia Medical Semi-Monthly.

The great majority of these casese were referred to me with the supposition of a primary bladder infection or cystitis. It is interesting that in only three of the fourteen cases was tuberculosis of the kidney primarily suspected.

I have been so impressed with the unreliability of symptoms that I am convinced it is only by a systematic examination of all infections of the urinary tract with reference to the causative organism that more diagnosis of renal tuberculosis may be made. The symptoms must not be depended on as a basis of diagnosis, and are of value, I believe, only to call attention to an infection of the urinary tract, the exact etiology of which has to be worked out in every case by painstaking and exact urinary examinations. Impressed as I am from a study of these histories of the minor importance of the symptoms from a diagnostic point, in this small paper, based principally on a study of these cases of renal tuberculosis, I have laid little stress on the symptoms as an aid to diagnosis.

The early recognition of few diseases is of such vital importance to the individual as that of renal tuberculosis. In spite of this, we are familiar with few infections where the diagnosis is made comparatively so late. In looking over the histories of these cases, I am impressed with the fact that after symptoms of the infection had appeared, there elapsed at least ten months before the earliest diagnosis of tuberculosis was made. In the great majority of cases the infection was well in the second year before the diagnosis was made. It is hardly necessary to recall that the importance of early diagnosis arises from the fact that the disease is almost always unilateral at first and later both kidneys are affected. Quoting Kelly and Burnham, "most every case which has a bilateral involvement and comes to autopsy shows an unequal involvement—a total involvement of one and a partial involvement of the other; indicating that in the early stage of the disease it is unilateral." In my rather small series of cases three showed a bilateral involvement—a double kidney infection. I wish to call attention, too, to the fact that practically all late cases show bladder and ureteral infection, which greatly complicates treatment. Thirty per cent. of these cases, as shown by statistics, are primary tuberculosis of the kidney, and when the diagnosis is made early and the kidney removed the great majority of these cases remain well; of this there can be no question.

About four years ago, Dr. Ralph Hamilton, Professor of Bacteriology of Georgetown University, first called my attention to the comparative ease and simplicity of demonstrating tubercle bacilli in the urine of cases of renal tuberculosis. Observing his remarkable successful demonstration of the organism in so many cases, I was influenced to try this method as a routine one in the examination of all kidney infections coming under my care. My cases have all convinced me that not only is the early diagnosis of renal tuberculosis practical, but it is rather easily made. For the early diagnosis of this infection of the kidney, I mainly depend now on the recognition of the organism in smears made from centrifuged specimens of urine. At first, I was impressed with the idea that it was necessary to use a very rapidly revolving centrifuge in order to throw down the bacilli, and about a year ago, when Crabtree wrote a paper giving specific direction for centrifuging the urine in these cases, I followed his technique for some months; lately, I have been impressed with the fact that almost any centrifuge will throw down enough bacilli to be easily recognized when stained specimens are made in the usual way of hunting for tubercle bacilli in the sputum. I have in many cases found the bacilli in the sediment of the urine allowed to simply gravitate in a sediment glass, yet, of course, I appreciate that the centrifuge is much more exact. Different methods of staining have been tried, but I think the old Gabbett method of heating carbol-fuchsin to steaming for three minutes on the smear and then counter-staining with acid methylene blue is as successful as any in my hand. As pointed out by Cabot and Crabtree, tubercle bacilli are always plentiful in urine from a kidney with tuberculosis, as is proven a priori by the fact that guinea pig inoculation with only a cubic centimeter of the uncentrifuged urine, is almost always successful in producing tuberculosis, showing that the tubercle bacilli are in such numbers that no concentration is necessary by centrifuge, etc. I am, of course well aware of some danger of the diagnosis of renal tuberculosis by smears made from centrifuged urine alone. I have myself come across two cases where men of large experience in bacteriological work have evidently mistaken the smegma bacillus for the tubercle bacillus. I am, however, thoroughly in accord with the observation of Crabtree, that the smegma bacillus is not present in the bladder urine except by contamination and is even less likely to be present in the catheterized urine obtained by ureteral catheterization. So that, though this is a possible danger, yet, when checked up by repeated examinations and the clinical findings, there is little possibility of this error being made.

Where there is the least doubt after careful cystoscopic examination and obtaining a specimen of urine from the infected kidney, I believe that guinea pig inoculation should be done and, if possible, done by the rapid methods suggested recently by J. J. Morton, of Boston. The guinea pig is first treated with the X-ray for several minutes. This appears to lower its resistance to the tubercle bacillus. A subsequent inoculation with the suspected urine will often be follow by tuberculosis in from seven to nine days. My own experience with this method has been very limited, but it appears to be a distinct advance in the rapid diagnosis of tuberculosis. I have, of course, cystoscoped all my cases and from the cystoscope have learned much valuable data, especially valuable in determining what operative procedure to take. I have been particularly impressed with the value of the cystoscope in determining the condition of the bladder, whether ulcerated or not; the probable condition of the ureter as indicated by the condition of the ureteral orifice; and in most cases the cystoscope was necessary to tell which kidney was affected, as the symptoms were inconclusive. It is a primary consideration, too, to learn the condition of the second kidney, if ther is one. (I have had one case of renal tuberculosis in one side of a horse-shoe kidney.) It is, of course, an important function of the cystoscopist to note the function of the separate kidneys after ureteral catheterization. All of these proceedings are of vital importance to the patient in the future handling of the case, but I insist that the general practitioner or the general surgeon who treats these conditions is not without responsibility on account of his inability to use the cystoscope and that he owes it to his patient and himself to examine carefully and exactingly a centrifuged specimen of the urine in every chronic urinary infection for the causative organism. I am certain that they will be able to find the organism and with little or no difficulty.

At the time of operation, after removal of the kidney, I have, as is usually done, sliced the kidney from pole to pole and have been impressed with the minuteness of the focus of infection in early cases. A patient may have the most intense bladder irritation and the urine be loaded with bacilli, yet the kidney when removed may show macroscopically the smallest foci of infection.

A study of my own and other specimens has indicated in the early cases an infection aroung the glomeruli, as pointed out by Baumgarten and others, and in none of the cases did there seem to be a focal infection in the pelvis of the kidney or in the lower medullary part of the kidney, though these parts are often secondarily infected. Hence, I believe that, though collargol injection of the pelvis of the kidney at the time the ureters are catheterized is of value in the advanced cases as a diagnostic aid, yet in the early cases the pelvis of the kidney has been changed so little that the X-Ray plate taken with Collargol in situ will show no recognizable change in the pelvis.

In the cases that I have been able to follow and whose urines I have examined for tuberculosis the diagnostic results have been very successful. In only about one in ten of these cases have I not been able to find tubercle bacilli in cases that have afterwards shown tuberculous infection. I have, of course, been compelled at times to make repeated examinations, but in the vast majority of these cases there was no difficulty in finding the organism.

I am thoroughly convinced that the ability of the observer to find the tubercle bacillus in a tubercular urine depends directly on the interest of the observer in the case. The cystoscopist who is thoroughly familiar with the clinical findings and has a direct interest in the case should do this himself. The laboratory man, however competent, knowing little of the case and having no direct interest in the case, will repeatedly pass over the examination and give a negative result. I have had any number of such reports. Careful, slow and painstaking examination of these suspected urines will show the tubercle bacillus in almost every case where there is a tubercular infection. As I examine more and more cases of tuberculosis of the kidney, I am fast becoming convinced that careful examination of the urine, as suggested, will show the tubercle bacillus in all cases.

In conclusion, I feel that every physician who treats urinary infection cannot longer be satisfied with the simple examination of the urine for pus and blood. He must in every case determine the causative organism present, and I am equally sure that he will not find this a difficult or onerous task if he will only develop the habit of looking for the cause of urinary infection. I am not unmindful of adding another burden to the already over-burdened practitioner of medicine in insisting on the examination of stained specimens of centrifuged urine in every case of infection of the urinary tract. but he who treats urinary affection must assume this burden.

We must in every case of urinary infection develop the habit of determining the organism causing the infection. We must get the habit of usuing the centrifuge more generally in all our urinary infections. We can with a little care and observation easily eliminate urethral and prostatic infections. In the absense of these, a catheterized speciment of urine is urgently demanded. This specimen in all probability contains the causative organism and, if centrifuged and the sediment examined, will show the organism in unmistakable numbers, whether it be the tubercle bacillus or other organism. At least two smears should be made of the centrifuged specimen, one to be stained with a simple stain for ordinary organisms, and the other to be stained for tubercle bacilli.

It is, I feel, by such routine examinations only that the practitioner will be enabled to make more diagnoses of renal tuberculosis and especially more early diagnoses.

